# Barrier Effects of the Kuroshio Current on the East Asian Northerly Monsoon: A Sensitivity Analysis

**DOI:** 10.1038/s41598-018-36577-9

**Published:** 2018-12-21

**Authors:** Jiayi Pan, Xue Feng, Wenfeng Lai, Adam T. Devlin, Hui Lin

**Affiliations:** 1 Institute of Space and Earth Information Science, The Chinese University of Hong Kong, Hong Kong, China; 2 School of Marine Sciences, Nanjing University of Information Science and Technology, Nanjing, Jiangsu China; 3 Shenzhen Research Institute, The Chinese University of Hong Kong, Shenzhen, Guangdong China

## Abstract

Through the use of satellite scatterometer data, it is observed that the East Asian northerly monsoonal winds decrease drastically when crossing the Kuroshio Current. In a section across the Kuroshio Current region, as revealed by reanalysis data, it is suggested that the upward velocity has a two-cell structure extending to the 500 hPa height, and a strong atmospheric convergence is present below the 900 hPa level. The reanalysis data also show that the northerly wind speed decreases significantly when crossing the Kuroshio Current region below 850 hPa height. A sensitivity analysis is implemented using the Weather Research and Forecasting (WRF) model, showing that the atmospheric convergence and the northerly wind drop are enhanced as the Kuroshio region surface temperature increases. This indicates that the Kuroshio Current can act as a barrier to the East Asian northerly monsoonal winds in winter.

## Introduction

Western boundary warm currents are observed to have significant effects on surface wind speed due to the strong sea surface temperature (SST) gradient^1–3^. Satellite scatterometer winds revealed that there are atmospheric convergences over the Kuroshio and the Gulf Stream regions^2,4^. The heat flux from the warm ocean currents may help to develop an unstable condition in the lower atmosphere^5,6^. These convective atmospheric flows transport surface moisture to a high level of the troposphere and increase the local precipitation^1,2,4^. Deep atmospheric convective activities over the warm tongue of the Kuroshio Current can be revealed by satellite data and the numerical modeling^7^. In the Gulf Stream region, higher precipitation is closely correlated with the atmospheric convergence, and the Gulf Stream can influence the entire troposphere^2^. In the rain band of the Gulf Stream, upward motion and cloud formation extend into the upper troposphere, as corroborated by the frequent occurrence of very low cloud-top temperatures^8^. Recent studies have reported climatological effects of the warm ocean currents. For instance, a narrow-band of precipitation over the Kuroshio in the East China Sea can lead to disastrous heavy rainfall events based on high-resolution satellite data, operational data, and model results^9^. Up to 25–30% of the variability of the summer precipitation in the Gulf Stream region is connected to the ocean surface boundary conditions^10^.

Previous investigations have shown strong correlations between SST and wind speed due to the fact that sea surface temperature can influence the atmospheric stability, which may in turn affect coupling between the surface winds and stronger winds aloft^2,3^. In the warm western boundary currents, there exist strong heat fluxes from the ocean to the atmosphere, especially in the winter season. Heat released from the warm Kuroshio Current provides energy for atmospheric convection over the warm current; this study further explores the effect of strong convection caused by the warm Kuroshio Current on the regional monsoon system, and identifies the role of the Kuroshio Current in adjusting regional atmospheric circulations.

## Surface Winds

Satellite scatterometer observations of ocean surface winds are used in this study, including the European Space Agency (ESA) ERS-1/2 scatterometer wind data (1° × 1° grid) and the National Aeronautics and Space Administration (NASA) QuikSCAT data (0.25° × 0.25° grid) available on the website of French ERS Processing and Archiving Facility of French Research Institute for Exploitation of the Sea (IFREMER, http://cersat.ifremer.fr/).

**Figure 1:**
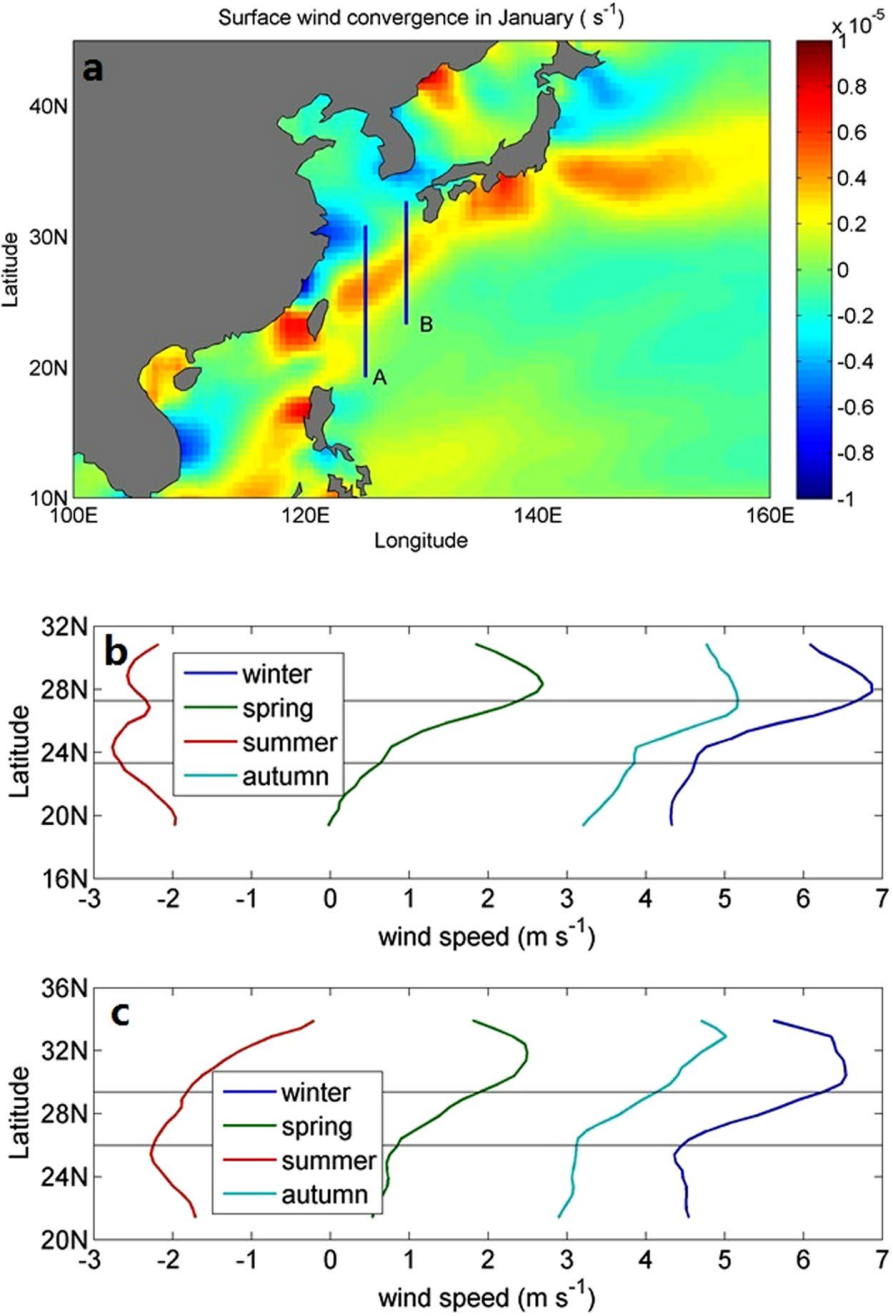
The convergence of meridional winds in January (1992 to 2000) (**a**) derived from ERS-1/2 winds and meridional wind speeds (NASA QuikSCAT scatterometer, with the positive values denoting the southward direction) across the Kuroshio during winter, spring, summer, and autumn (in 2002–2008) along A(**b**) and B (**c**). The latitude range between the solid lines shows the Kuroshio Current region.

Figure 1a shows the mean atmospheric convergence of the meridional wind in January. The wind convergence is derived from the ERS-1/2 wind data in January from 1992 to 2000, and the mean convergence in January is obtained by averaging all the January data from 1992 to 2000. This convergence has a prominent annual cycle, which is stronger in winter and weaker in summer. Over the East China Sea, the surface winds are controlled by the East Asian monsoon with southerly winds in summer and northerly winds in winter^11,12^. In winter, dry northerly winds prevail all the way to the Kuroshio region. Figure 1b displays the sea surface meridional wind speed from the QuikSCAT winds along two sections (A and B in Figure 1a) in winter, spring, summer, and autumn (positive values are defined as southward), which are obtained from the seasonal means of monthly wind data from 2002 to 2008. The wind speed decreases substantially after crossing the Kuroshio Current region in winter, spring, and autumn when the northerly monsoonal winds prevail. For section A (B), the wind speeds are reduced by 2.2 (2.2), 1.8 (1.6), and 1.4 (1.4) m s^−1^ in winter, spring, and autumn, respectively. The observed decreases in wind speed are likely related to the warm Kuroshio Current, which may serve as a barrier to the winter monsoonal winds and block the southward transport of dry and cold air mass from the high latitude area. In the following section, the vertical structure of the atmosphere is analyzed to determine the influence of the high temperature Kuroshio Current on the northerly monsoonal winds.

### Vertical Structure

**Figure 2:**
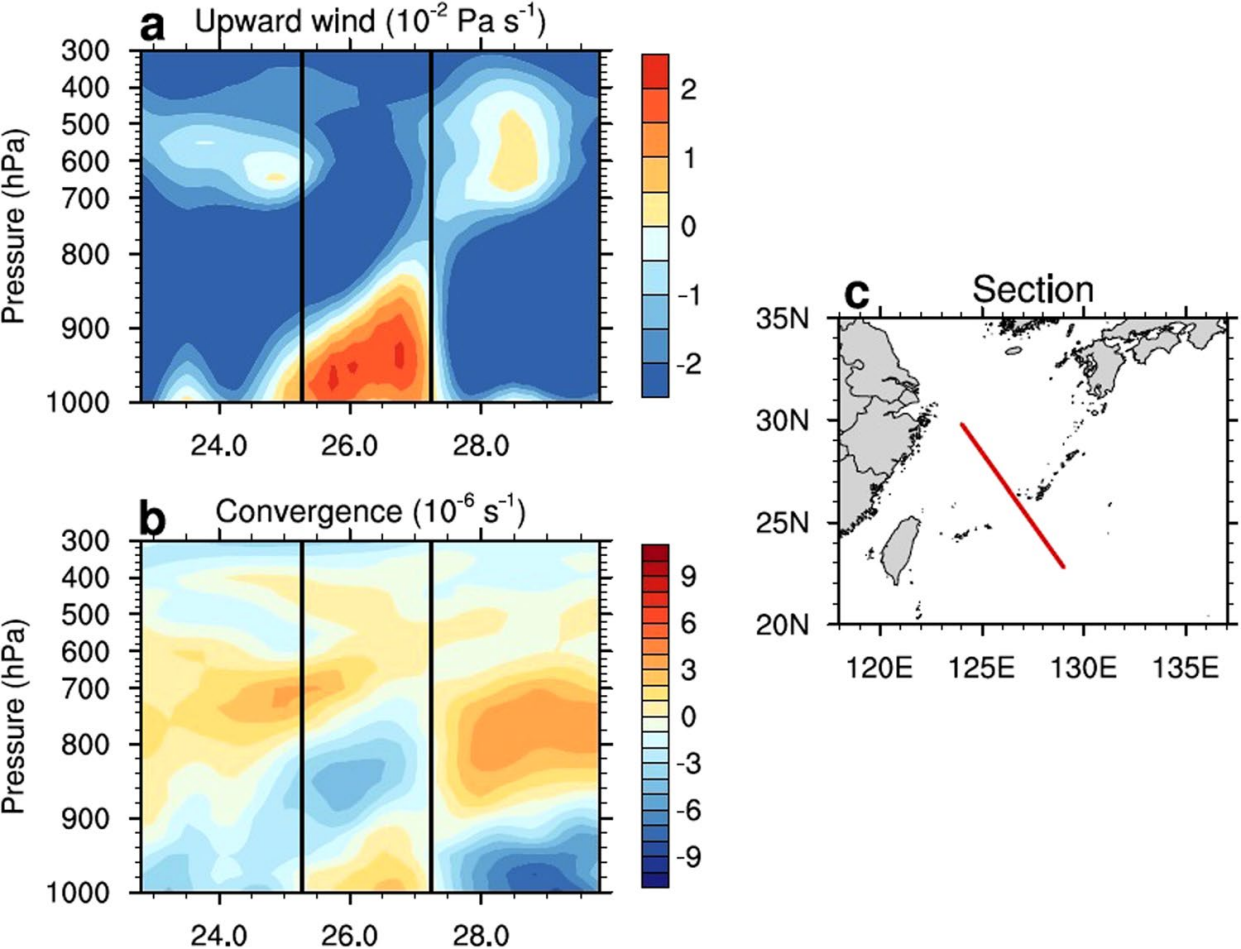
Vertical profiles of the vertical velocity (**a**) and wind convergence (**b**) across the Kuroshio Current along a section shown in (**c**). The ranges in (**a**) and (**b**) enclosed by the black lines show the Kuroshio Current region.

The vertical structure of the winds over the Kuroshio region is displayed using the era-interim monthly mean product from the European Center for Medium-Range Weather Forecasts (ECMWF) Center. The era-interim product has 37 pressure levels from 1000 to 1 hPa at a horizontal resolution of 0.25° × 0.25°. Our analysis is based on the January mean of the winds from 2005 to 2008. Figure 2 shows the vertical profiles of the vertical velocity (a) and wind convergence (b) across the Kuroshio Current along a section shown in (c). An upward velocity (*w* > 0) appears over/near the Kuroshio Current region up to a pressure level of 300 hPa. The upward winds have two cells with a maximum velocity located around 960 hPa and 600 hPa; the formation of these two cells is due to two kinds of dynamic mechanisms for the upward motion of air masses. First, a low level upward motion is caused by the sensible heat flux near the ocean surface; second, the upper cell is driven by buoyancy resulting from the latent heat released during high precipitation over the Kuroshio region^3^. Figure 2b shows that below the 900 hPa level exists a cell of positive convergence, indicating the direct influence of the Kuroshio Current. In addition, an upper convergence cell appears on the right side of the Kuroshio Current region with a larger size than the lower cell. In the lower level at a pressure less than 900 hPa, the sensible heat dominates the total heat fluxes; in the high level (>900 hPa), the sensible heat drops significantly, and the latent heat increases and is much higher than the sensible heat, reaching the maximum at the 600 hPa^7^. These vertical profiles of the heat fluxes suggest that the sensible heat only affects the lower level of the atmosphere and in the mid-level (higher than 900 hPa), the latent heat takes effect.

**Figure 3:**
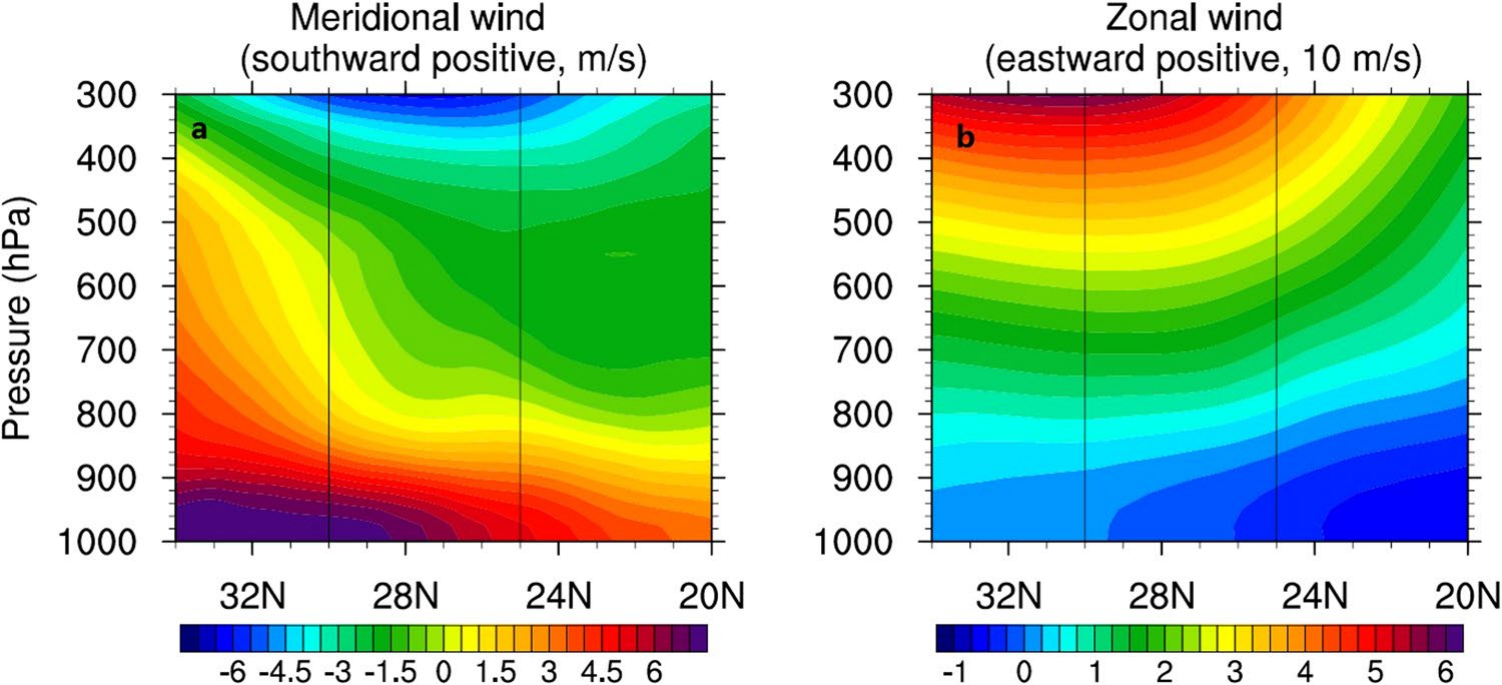
Vertical profiles of meridional northerly (**a**) and zonal (**b**) winds along the section in Figure 2c. The positive values denote the southward direction. The latitude range between the vertical lines shows the Kuroshio Current region.

The vertical views of meridional northerly (a) and zonal (b) winds along the section (illustrated in Figure 2c) are shown in Figure 3 (with positives values denoting a southward direction). The Kuroshio Current region is also marked in the figure. Consistent with the scatterometer surface winds, the ECMWF meridional winds in the lower level exhibit a sharp drop when crossing the Kuroshio Current region below the height of 850 hPa, corresponding to a strong atmospheric convergence below that level. In this lower layer, the zonal winds are relatively weak compared with the northerly winds Figure (3b) and change from the westerly to the easterly. Figure 3 reveals that the winter northerly winds mainly exist in the lower level below the 800 hPa height. The ECMWF data indicate that the winter northerly monsoonal winds are significantly reduced over the Kuroshio Current region in the lower level where the atmospheric convergence is strong, revealing the barrier effect of the Kuroshio Current-induced atmospheric convection on the winter northerly winds.

### Sensitivity analysis

**Figure 4:**
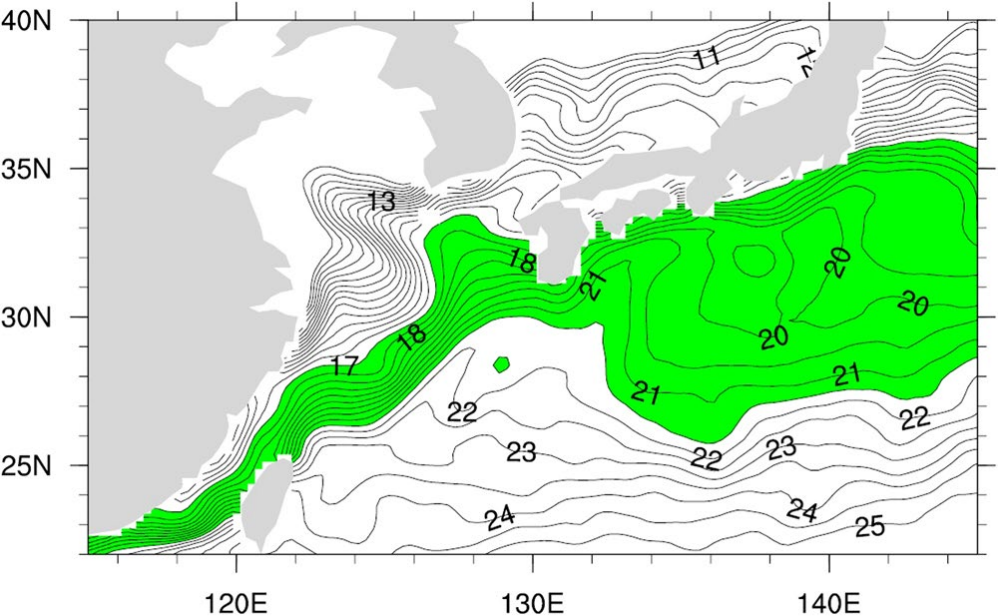
Sea surface temperature with SST 17.0~21.5 °C in green color.

The influence of the Kuroshio Current on the regional atmospheric circulation can be investigated by a sensitivity analysis that adjusts the Kuroshio region surface temperature. The Weather Research and Forecasting (WRF) model is used to examine the sensitivity, with 40 sigma layers in the vertical. The model initial and boundary conditions are obtained from Final Analysis (FNL) and Tropospheric Analyses products of the National Centers for Environmental Prediction (NCEP), available on a 1° × 1° grid with 26 vertical pressure levels from 1000 to 10 mbar. For the lower boundary condition of the WRF model, SST data are taken from the National Climatic Data Center (NCDC) AMSR + AVHRR optimally interpolated data on a 1/4° grid. A nesting domain method is used, with the larger domain covering 19°−40°N, 114°−160°E and a small, inner domain covering 20.3°−36.5° N, 118°−145° E. The outer domain (30 km grid) provides lateral boundary conditions for the inner domain (10 km grid) in model operation. We first run the simulation using a realistic SST, and then we perform two experiments with the SST in the Kuroshio Current region (SST 17.0~21.5 °C, as shown in Figure 4, where the SST gradient is high) multiplied by 1.2 and 1.4, respectively.

**Figure 5:**
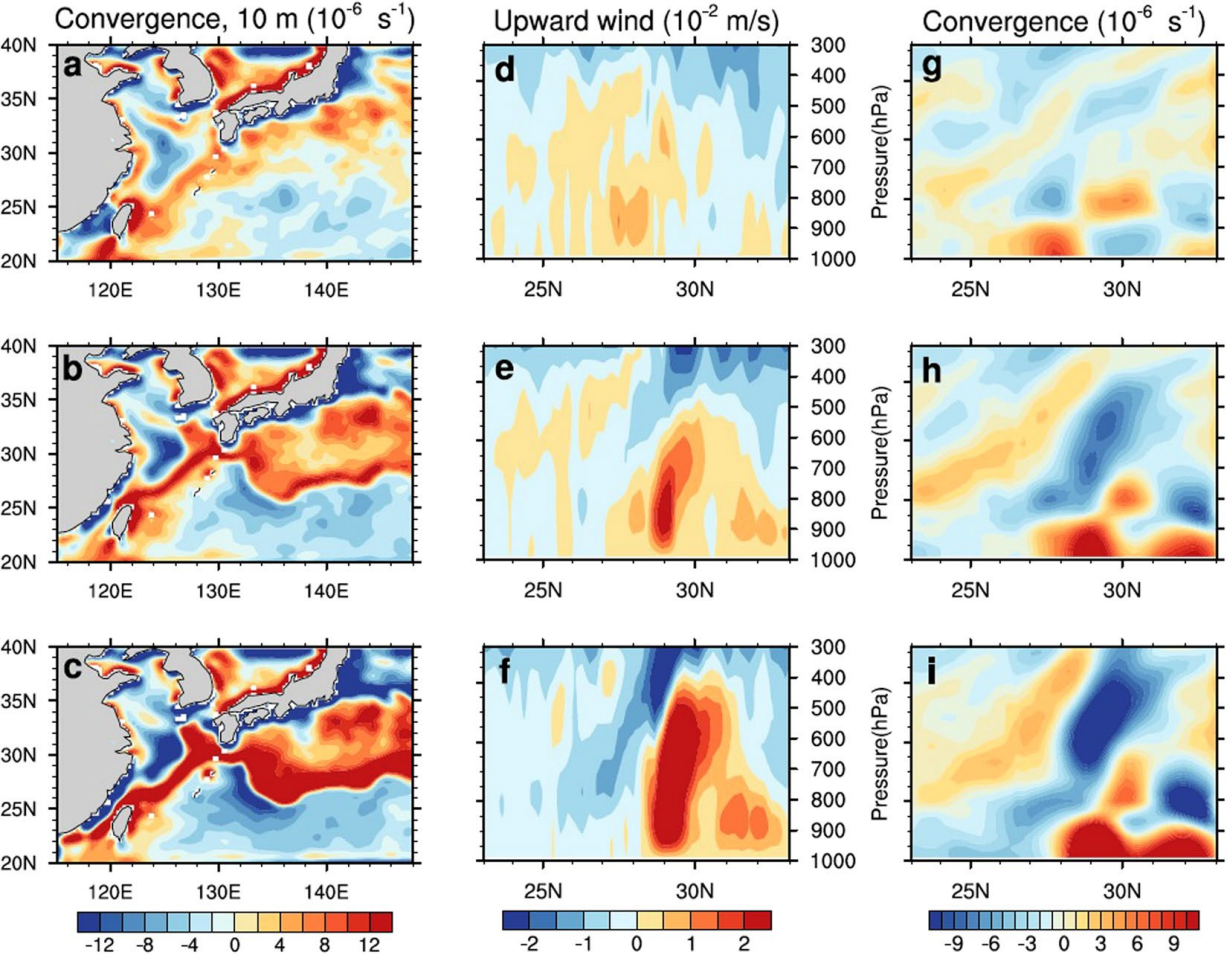
WRF model sensitivity analysis results: surface convergence, sectional vertical velocity and convergence along 127 °E for the Kuroshio SST multiplied by 1.0 (**a**,** d**, and **g**), 1.2 (**b**, **e**, and **h**), and 1.4 (**c**, **f**, and **i**).

Figure 5 reveals the surface wind convergence and profiles of the vertical velocity and convergence along 127 °E. As the Kuroshio Current temperature increases, the surface atmospheric convergence is enhanced (Figures 5a–5c), and increases by 2.0 and 3.2 times as the Kuroshio SST is amplified by 1.2 and 1.4, respectively. The upward speed becomes high in response to the Kuroshio SST increase (Figures 5d–5f). The wind convergence and upward wind are enhanced in the SST increase region (as shown in Figure 4). Consistent with the ECMWF data, the relatively strong upward speed from the WRF model exists below the height of 900 hPa; the strong upward wind cell extends up to the 500 hPa level as the Kuroshio SST increases 1.4 times (Figure 5f). The convergence along 127 °E also increases with a rise in the Kuroshio SST (Fig. 5g–i). At the 900 hPa level, the convergence increases by 4.7 and 8.2 times as the Kuroshio SST is multiplied by 1.2 and 1.4, respectively. Additionally, the strong convergence may extend to the 700 hPa level under the highest SST increase scenario Figure (5i).

**Figure 6:**
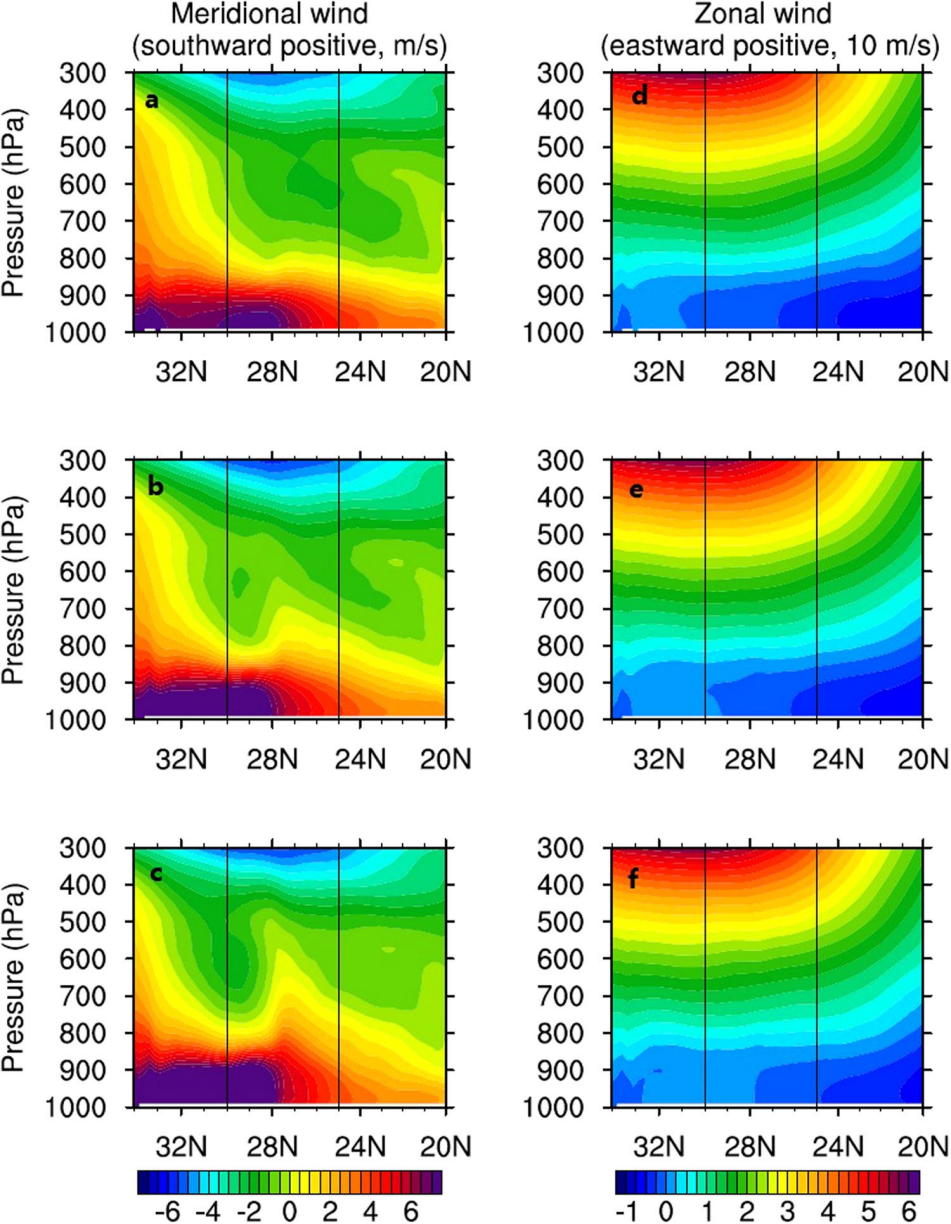
Vertical profiles of meridional northerly (left) and zonal (right) winds along the meridional section of 127 °E under the scenarios of the Kuroshio SST multiplied by 1.0 (**a**,** d**), 1.2 (**b**,** e**), and 1.4 (**c**,** f**). The positive values denote the southward direction. The latitude range between the vertical lines indicates the Kuroshio Current region.

The horizontal velocities along the meridional section of 127° E versus the varied Kuroshio Current temperatures are shown in Figure 6. Figures 6a–6c illustrate the meridional velocities corresponding to the Kuroshio Current temperature multiplied by 1.0, 1.2, and 1.4, respectively, and Figures 6d–6f reveal zonal velocities with the Kuroshio Current temperature multiplied by 1.0, 1.2, and 1.4, respectively. The general patterns of wind speed variation in the Kuroshio Current region are consistent with the results of ECMWF data analysis; an abrupt drop occurs in the lower atmospheric level in the meridional direction. When the Kuroshio SST increases, the decrease in the meridional wind speed is stronger. Table 1 lists the northerly wind speed drop in the lower level from 30 °N to 25 °N along 127 °E. The table indicates that the wind speed decreases as much as 6.2 m s^−1^ while the Kuroshio temperature is amplified by 1.4 times at the 950 hPa height, above which height, the meridional winds also exhibit substantial reduction. When the Kuroshio temperature is amplified by 1.4, the pressure level where the northerly winds exhibit abrupt drops reaches 800 hPa. Although the zonal winds are weak, Figures 6d–6f reveal that the zonal winds do not change much compared with the meridional winds as the Kuroshio temperature rises. The sensitivity analysis suggests the atmospheric convection over the Kuroshio becomes stronger with increasing Kuroshio SST, this enhanced convection can further decrease the winter northerly wind, and therefore, the barrier effects of the Kuroshio-induced atmospheric convection on the winter northerly winds become stronger with the higher Kuroshio SST.

**Table 1:** Meridional northerly wind speed decrease in the southward direction across the Kuroshio region from 30 °N to 25 °N along 127 °E.

Kuroshio SST	975 hPa (m s^−1^)	950 hPa (m s^−1^)	925 hPa (m s^−1^)	900 hPa (m s^−1^)	850 hPa (m s^−1^)
1.0×	3.0	2.8	2.5	2.0	0.9
1.2×	4.4	4.3	4.0	3.4	1.1
1.4×	6.2	6.0	5.7	5.0	2.2

## Discussion and Summary

Western boundary current regions such as the Kuroshio have significant heat fluxes that are the main energy source for strong atmospheric convection. Model experiments show that sensible and latent heat fluxes increase with the Kuroshio SST rise (not shown). In the Kuroshio Current region, the direct influence of the ocean sensible heat flux is at the lower level, and in the mid-level (750-500 hPa), the latent heat released from the precipitation is a main energy source for the upward atmospheric motion^7^. This study shows that in the surface layer, the northerly monsoonal wind in winter has a significant drop in the lower layer as a result of the direct sensible heating. In the northwest Atlantic, the influence of the Gulf Stream may extend to the top of the troposphere^5^. The Kuroshio Current covers an area with distinctly different atmospheric background circulation patterns from its Gulf Stream counterpart. This may be one of the reasons for the difference in the atmospheric response to SST observed in the Kuroshio and Gulf Stream regions.

There are a chain of islands exists in the Kuroshio Current region. Comparing the lateral scales of the island and upward motion, one can find that the lateral scale of the upward motion has a range of 4° at latitudes (400 km), while the lateral scale of the island chain is much smaller, as shown in Figure 2a. Thus, the islands cannot provide much heat energy to support the upward motion on such a large scale. A previous modeling study also validated that the small scale islands do not have much effect on the Kuroshio region atmosphere^7^. Therefore, it is plausible that the islands cannot cause this upward motion in this vast range.

This study used satellite scatterometer winds and ECMWF reanalysis data to analyze the influence of the atmospheric convection over the high temperature Kuroshio Current on the East Asian northerly monsoonal winds. Satellite scatterometer observations show that the northerly surface winds over the East China Sea exhibit a substantial drop when crossing the Kuroshio Current region. This phenomenon is also revealed in the ECMWF wind data. In a section across the Kuroshio Current, there are two cells of upward velocity observed: one is below the 900 hPa level and the other is between 750 to 400 hPa. Additionally, the Kuroshio-induced atmospheric convergence may extend to a height of 900 hPa. The horizontal wind velocity drops sharply within the lower level (>850 hPa) in the meridional direction, associated with the upward velocity (same as that at the surface). A sensitivity analysis based on the WRF model further confirms our results. When the Kuroshio Current SST increases, the Kuroshio Current-induced atmospheric convergence and upward velocity strengthen, and the horizontal northerly wind velocity in the lower level exhibits a much sharper decrease when crossing the Kushiro Current region. This indicates that the atmospheric convection blocks the northerly monsoonal winds and may affect the regional atmospheric circulation.
